# Rupture and intra-peritoneal bleeding of a hepatocellular carcinoma after a transarterial chemoembolization procedure: a case report

**DOI:** 10.1186/1757-1626-2-68

**Published:** 2009-01-20

**Authors:** Artan Reso, Chad G Ball, Francis R Sutherland, Oliver Bathe, Elijah Dixon

**Affiliations:** 1 University of Calgary, Department of Surgery, Foothills Hospital, 1403-29 St N.W, T2N 2T9, Calgary, Alberta, Canada

## Abstract

**Background:**

Transarterial chemoembolization (TACE) is a well accepted treatment for inoperable hepatocellular carcinoma (HCC). While minor complications involve 10% of all patients, severe complications are rare.

**Case Presentation:**

We describe a case of a 90-year-old male with a large, superficial HCC who underwent TACE. He had a significant intraperitoneal bleed secondary to tumor rupture immediately following the procedure.

**Conclusion:**

Tumor size and superficial location must be considered risk factors for tumor rupture and related complications.

## Introduction

Although operative resection is the preferred method of treatment for hepatocellular carcinoma (HCC), only 20% of patients are candidates for resection at the time of diagnosis [1,2]. As a result of its therapeutic successes, survival advantage and minimally invasive technique, transarterial chemoembolization (TACE) has become a well accepted treatment for inoperable HCC [1,3,4]. While the specific agents employed in TACE are varied, a reduction in hepatic arterial blood supply to the tumor, as well as the delivery of tumorcidal agents, remain the basic principles.

Numerous publications describe minor complications associated with TACE in 10–12% of patients [2-8]. These include postembolization syndrome (fever, abdominal pain, nausea, and vomiting), impaired liver function, and leukocytopenia [2,3,6]. While these are common, there are very few reports of severe post-TACE complications in the literature [6-8]. Major complications include a 3% rate of irreversible liver failure, as well as liver abscess, upper gastrointestinal bleeding, bile duct complications, acalculous cholecystitis, pulmonary embolism, spasm or occlusion of hepatic artery and acute renal failure.

## Case presentation

**Figure 1:**
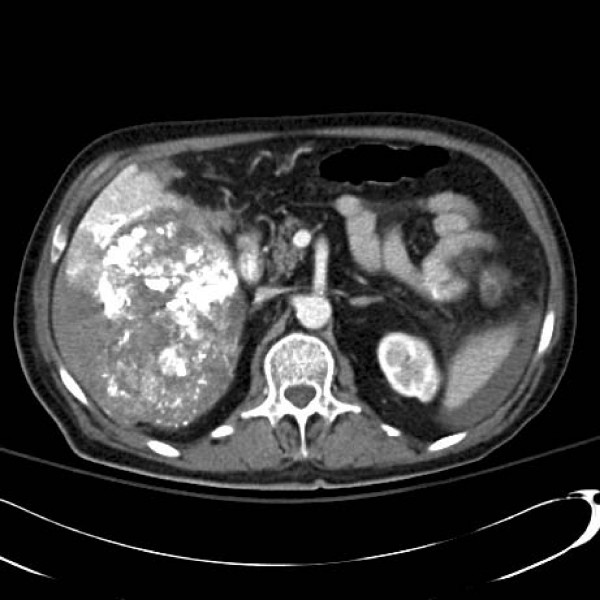
**Computed tomographic scan of ruptured hepatocellular carcinoma post-TACE**.

**Figure 2:**
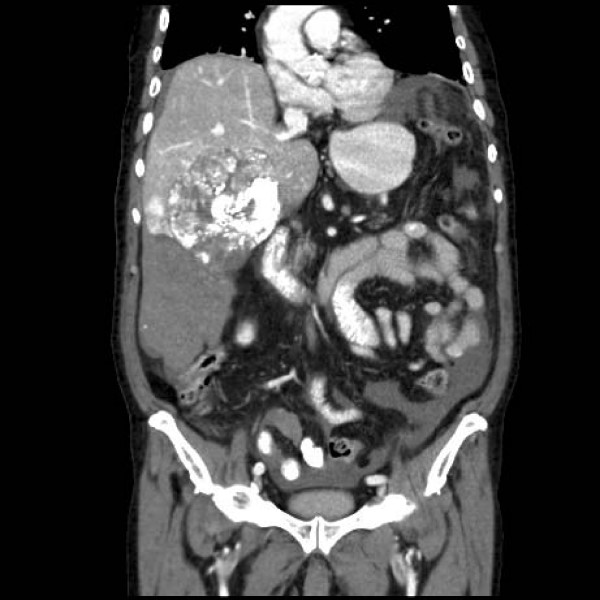
**Computed tomographic scan of ruptured hepatocellular carcinoma with associated hemorrhage immediately post-TACE**.

A 90-year-old male with a history of hypertension and diabetes was incidentally diagnosed with a HCC. CT described a large and hypervascular mass in the right hepatic lobe (10 × 11 × 7 cm) in close proximity to the liver capsule. His liver enzymes and function tests were normal. He underwent transarterial chemoembolization (TACE) with no intra-operative complications. The tumor was embolized with a combination of 50 mg of Cisplatin, 50 mg of Adriamycin and 20 mL of Lipidol. Within 4 hours of the procedure however, the patient became hypotensive (systolic blood pressure = 90 mmHg), tachycardic (heart rate = 156 beats per minute) and developed a moderate abdominal tenderness. His hemoglobin decreased from 141 to 92 g/L. The patient stabilized with 1.5 liters of crystalloid resuscitation. An urgent CT scan revealed a large hemoperitoneum with no active arterial contrast extravasation (Figures 1 &2). Scattered punctuate foci of hyperattenuated particles located in the right paracolic gutter suggested free intra-peritoneal chemoembolization agents. In spite of the ruptured HCC, the patient underwent conservative management without a laparotomy. He required 2 units of packed red blood cells and 4 units of fresh frozen plasma after developing progressive anemia, post-embolization syndrome and mild liver impairment. He was also given intravenous antibiotics (ceftraixone and flagyl). He recovered from his intra-abdominal hemorrhage, but eventually died of ongoing respiratory failure on post-TACE day 16.

## Discussion

Our patient displayed a ruptured hepatocellular carcinoma almost immediately after undergoing TACE. Rupture, and its associated hemorrhage, represents a very rare complication. The mechanism of bleeding is likely related to necrosis of the liver capsule as a result of the chemoembolization agents. The patient's ability to tolerate this hemorrhage was likely limited by his age. There have only been 5 cases reported in the literature [6,7]. As a collective, these reports offer tumor size and superficial location as possible risk factors for rupture. Four of five patients also appeared to rupture in a relatively delayed timeframe (2 to 45 days post TACE). Only 1 patient, in addition to ours, became symptomatic immediately after the procedure [6]. Furthermore, our patient was the only case who did not undergo a laparotomy.

## Conclusion

Although TACE is generally a safe procedure, intraperitoneal bleeding due to tumor rupture must be considered a potential complication when patients respond poorly immediately after the procedure. As exemplified in our patient, this appears especially important in large tumors located adjacent to the liver capsule.

## Patient family's perspective

"This beloved man was a caring person who's intellectual abilities were entirely normal before the procedure. He understood his disease and the treatment options offered to him. At the time he was diagnosed, he was leading a good life, was happy and enjoyed himself. He was committed to pursuing any and all options to fight the disease, at any cost. Close family members had died from cancer before him with basically no treatment and immense suffering. He was determined to go ahead with any treatment that could help him live longer or relieve symptoms."

## Competing interests

The authors declare that they have no competing interests.

## Authors' contributions

AR and CGB collected and analyzed all patient data. AR, CGB, FRS, OB, and ED each assisted in writing the manuscript, as well as with critical revision. All authors read and approved the final manuscript.

## Consent

"Written informed consent was obtained from the patient's wife for publication of this case report and accompanying images. A copy of the written consent is available for review by the Editor-in-Chief of this journal."
